# Prevalence and factors associated with depression, anxiety and stress among nursing home caregivers in China: a multi-center cross-sectional study

**DOI:** 10.3389/fpubh.2025.1690840

**Published:** 2025-11-03

**Authors:** Zhi-Ru Fan, Mei Chan Chong, Zheng Xiaona, Ma Jing, Yan Li You, Lili Ma, Chong Chin Che

**Affiliations:** ^1^Department of Nursing Science, Faculty of Medicine, Universiti Malaya, Kuala Lumpur, Malaysia; ^2^Department of Physiology, North Henan Medical University, Xinxiang, China; ^3^Department of Geriatric Medicine, Shengli Clinical Medical College of Fujian Medical University, Fuzhou, China; ^4^Fujian Provincial Center for Geriatrics, Fuzhou University Affiliated Provincial Hospital, Fuzhou, China; ^5^School of Nursing, Henan University of Science and Technology, Luoyang, Henan, China; ^6^Rheumatology Unit, The First Affiliated Hospital, College of Clinical Medicine of Henan University of Science and Technology, Luoyang, China

**Keywords:** nursing home caregivers, depression, anxiety, perceived stress, long-term care

## Abstract

**Background:**

This study aimed to investigate the prevalence of depression, anxiety, and perceived stress among nursing home caregivers in China and to examine the associated factors.

**Methods:**

A multicenter cross-sectional study was conducted among 1,341 caregivers in nursing homes across Henan Province, China. Standardized instruments were applied, including the Self-Rating Depression Scale (SDS), Self-Rating Anxiety Scale (SAS), and the 10-item Perceived Stress Scale (PSS-10). Descriptive statistics, univariate analysis, and multivariable logistic regression were performed to identify independent predictors of psychological distress.

**Results:**

The prevalence of depression and anxiety was 34.8 and 10.8%, respectively, while 49.6% of caregivers reported moderate-to-high levels of stress. Significant predictors included city region, type of nursing home, educational level, monthly income, working hours, night shifts, presence of chronic diseases, attention to mental health, and participation in psychological training. Higher education and moderate income were protective factors, whereas employment in rural private nursing homes, low engagement in mental health practices, and the presence of chronic diseases increased risks. Longer working hours and more frequent night shifts were unexpectedly associated with lower stress levels.

**Conclusion:**

Depression and stress represent the major psychological concerns among nursing home caregivers in China. Targeted interventions should prioritize routine mental health screening, workplace-based psychological support, and policy measures aimed at improving working conditions and access to training, thereby safeguarding caregiver well-being and supporting the sustainability of long-term care services.

## 1 Introduction

The rapid acceleration of global population aging has become one of the most pressing public health and social challenges of the 21st century (1). China, which accounts for one-fifth of the world’s older population (2), had 220 million people aged 65 years or above by the end of 2024, representing 15.6% of the total population (3). This number is projected to reach 366 million by 2050 (4). Such unprecedented demographic shifts have greatly intensified the demand for long-term care services and imposed considerable pressure on professional caregivers (5). Caregiving, whether provided formally or informally, involves substantial physical, emotional, and social demands, often leading to fatigue, stress, and burnout among care providers (6). These challenges are particularly pronounced in institutional settings where care needs are continuous and complex.

Nursing home caregivers play a crucial role in providing physical, emotional, and social support for older adults, particularly those who are frail, disabled, or living with chronic illnesses (7). However, caregiving in nursing homes is characterized by heavy physical workload and high emotional demands, while a global shortage of long-term care workers has become increasingly severe (8). In China, the long-term care sector faces substantial challenges. Nursing home caregivers often endure intense workloads, time pressure, and limited resources (9), alongside insufficient training, low wages, and limited social recognition (10, 11). These unfavorable conditions may lead to significant psychological distress (12).

Evidence suggests that nursing home caregivers represent a high-risk yet often overlooked group in terms of mental health (13). A systematic review by Gray, Birtles (14) reported that more than 20% of nursing home caregivers experienced anxiety and depressive symptoms, which was markedly higher than the prevalence among hospital nurses (15). A cross-sectional survey in northeastern China found that 44% of caregivers reported anxiety and 19.4% reported depressive symptoms (12). Studies from Germany, France, Spain, and Japan also indicated high prevalence rates of anxiety and depression among caregivers in long-term care facilities (16–20). In addition, perceived stress, defined as the individual’s subjective appraisal of external stressors, has been recognized as a key construct for understanding caregivers’ mental health (21). Elevated levels of perceived stress have been strongly linked to negative outcomes such as sleep disturbances, emotional exhaustion, somatic complaints, and health-damaging behaviors (20, 22).

Anxiety, depression, and perceived stress among nursing home caregivers are influenced by multiple demographic, occupational, and psychosocial factors. Individual characteristics, such as older age, lack of marital support, and poor health, have been shown to be associated with higher psychological distress (23–25). Occupational stressors, including heavy workload, low salary, shift work, and limited organizational support, also contribute substantially to emotional strain in long-term care (26–28). Night shifts and irregular work schedules may disrupt circadian rhythms, thereby increasing risks of depression, sleep problems, and somatic symptoms (12, 29).

Although awareness of caregivers’ mental health burden is growing, research in China remains limited, particularly large-scale, multicenter investigations that not only document the prevalence of psychological symptoms but also identify associated sociodemographic and occupational factors. To our knowledge, no prior study has systematically examined the comorbidity of anxiety, depression, and stress among Chinese nursing home caregivers. Identifying modifiable risk and protective factors is essential for developing targeted interventions, policy responses, and support mechanisms for caregivers. Therefore, the present study aimed to: (1) assess the prevalence of anxiety, depression, and perceived stress among nursing home caregivers in China; and (2) identify the risk and protective factors associated with these mental health outcomes.

## 2 Methods

### 2.1 Samples and data collection

**Figure 1:**
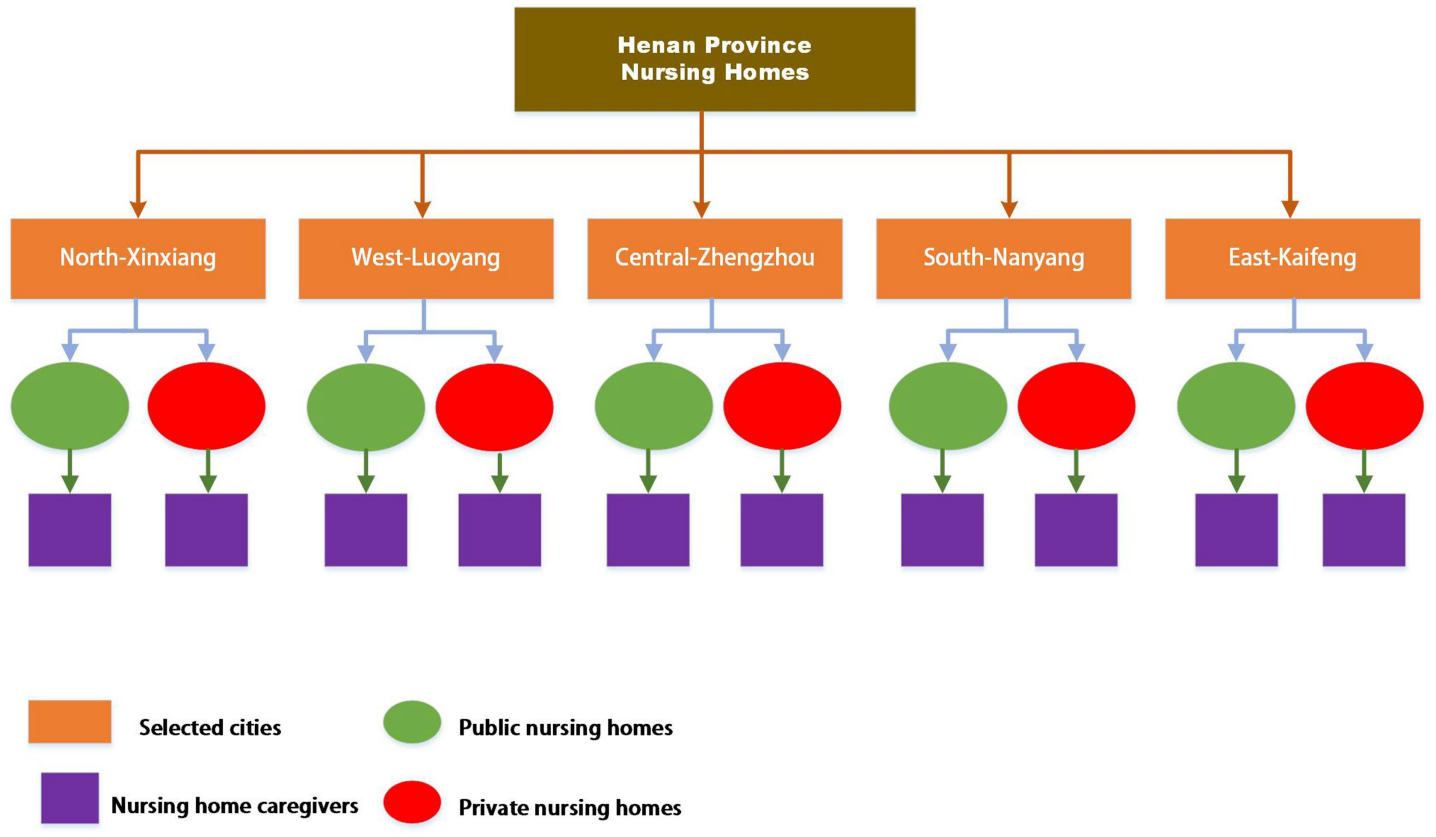
Stages of multi-stage sampling.

This multicenter cross-sectional study was conducted among nursing home caregivers in Henan Province, China, between November 2024 and February 2025. To ensure representativeness, a multi-stage cluster sampling strategy was applied. In the first stage, stratified random sampling was performed at the city level. The 18 prefecture-level cities in Henan Province were categorized into five geographic regions (north, south, east, west, and central). One city was randomly selected from each region: Zhengzhou, Xinxiang, Luoyang, Nanyang, and Kaifeng. In the second stage, public and private nursing homes were identified in each selected city, and disproportionate stratified sampling was used to select a total of 46 facilities (18 public and 28 private). In the third stage, eligible caregivers were recruited from the selected facilities using convenience sampling. The multi-stage sampling procedure is illustrated in Figure 1.

The required sample size was estimated according to the formula by Chow, Shao (30), with a significance level of *α* = 0.05 (95% confidence). When the allowable error (*δ*) was set at 0.05 and the estimated standard deviation (*σ*) was 0.83, the sample size was calculated as:


$$n={\left(\frac{Z_{\alpha /2}\cdot \sigma }{\delta }\right)}^{2}={\left(\frac{1.96\cdot 0.83}{0.05}\right)}^{2}\approx 1059$$

To account for a 20% potential non-response or sampling bias (31), the final target sample was 1,324 (1,059 ÷ 0.8 ≈ 1,324). The final valid sample of 1,341 caregivers exceeded this target, ensuring adequate statistical power and representativeness.

Inclusion criteria were: (1) currently employed as a caregiver in a nursing home, and (2) having worked at the institution for at least 6 months. Caregivers who were on leave or declined participation were excluded. With the support of institutional administrators, the research team first obtained formal permission from nursing home directors to conduct the survey (see Supplementary material), obtained the complete list of caregivers, and coordinated survey schedules. The study purpose, procedures, and confidentiality assurances were explained to all potential participants, and informed consent was obtained. Caregivers then voluntarily completed a structured questionnaire via the secure online platform Wenjuanxing, ensuring both convenience and data integrity.

The research team consisted of one doctoral student, one psychologist, and four trained data collectors. All team members received systematic training on study procedures, ethical principles, and participant support. Written permission was obtained from all participating institutions prior to data collection. Survey administration was scheduled in coordination with caregivers’ working hours to minimize disruption to daily care tasks.

### 2.2 Instruments

#### 2.2.1 Sociodemographic questionnaire

A structured questionnaire was used to collect sociodemographic characteristics, including gender, age, and years of work experience, among others.

#### 2.2.2 Self-rating anxiety scale (SAS)

Anxiety symptoms were assessed using the Self-Rating Anxiety Scale (SAS) developed by Zung (32) and translated and validated into Chinese by Wang, Zhengyu (33). The scale contains 20 items rated on a 4-point scale (“none or a little of the time” = 1 to “most or all of the time” = 4). Fifteen items are positively worded, and five (items 5, 9, 13, 17, and 19) are reverse scored. The raw total score is multiplied by 1.25 to yield a standardized score ranging from 25 to 100. Scores <50 indicate no anxiety, 50–59 mild anxiety, 60–69 moderate anxiety, and ≥70 severe anxiety. In this study, Cronbach’s *α* was 0.807.

#### 2.2.3 Self-rating depression scale (SDS)

Depressive symptoms were measured using the Self-Rating Depression Scale (SDS) developed by Zung and translated into Chinese by Wang, zhengyu (34). The SDS consists of 20 items rated on a 4-point scale (1 = rarely to 4 = most of the time). Ten items are positively worded, while items 2, 5, 6, 11, 12, 14, 16, 17, 18, and 20 are reverse scored. The raw score is multiplied by 1.25 to yield a standardized score (range: 25–100), with higher scores indicating greater severity of depression. Scores <50 indicate no depression, 50–59 mild, 60–69 moderate, and ≥70 severe depression (35). In this study, Cronbach’s *α* was 0.850.

#### 2.2.4 Perceived stress Scale-10 (PSS-10)

Perceived stress was measured using the 10-item Perceived Stress Scale (PSS-10) developed by Cohen, Kamarck (36) and translated into Chinese by Ng (37). The scale assesses the degree to which life situations are perceived as unpredictable, uncontrollable, and overwhelming over the past month. Items are rated on a 5-point Likert scale ranging from 0 (never) to 4 (very often), yielding a total score of 0–40. Scores of 0–13 indicate very low stress, 14–18 low to moderate stress, 19–25 moderate to high stress, and 26–40 high stress. Items 4, 5, 7, and 8 are reverse scored. In this study, Cronbach’s *α* was 0.775.

### 2.3 Statistical analyses

All statistical analyses were performed using SPSS version 26.0. Descriptive statistics were used to summarize the characteristics of the sample. Continuous variables were expressed as means and standard deviations (SD), while categorical variables were presented as frequencies and percentages. To examine differences in anxiety, depression, and perceived stress across sociodemographic groups, univariate analysis was conducted, with independent-samples t tests and one-way analysis of variance (ANOVA) used for continuous variables.

Multivariable logistic regression models were employed to identify independent factors associated with anxiety, depression, and perceived stress. Variables that were significant in the bivariate analyses were entered into the regression models. Odds ratios (ORs) with 95% confidence intervals (CIs) were reported. All tests were two-tailed, and a *p*-value < 0.05 was considered statistically significant.

Prior to multivariable logistic regression analyses of SAS, SDS, and PSS-10, collinearity diagnostics confirmed no multicollinearity (VIFs < 2), and Hosmer–Lemeshow tests indicated good model fit for all outcomes (SAS: χ^2^ = 9.226, *p* = 0.324; SDS: χ^2^ = 9.334, *p* = 0.315; PSS: χ^2^ = 6.987, *p* = 0.538), supporting robust assessment of sociodemographic and work-related influences on psychological distress, see Supplementary Tables S1–S6.

## 3 Result

### 3.1 Descriptive information

The online survey was validated on the Wenjuanxing platform prior to data download. Of the 1,500 distributed questionnaires, 147 were excluded due to incomplete responses, resulting in a final sample of 1,341 nursing home caregivers (response rate: 89.4%). The majority were women (75.6%), aged 51–60 years (46.6%), and married (90.5%). Most caregivers had a junior high school (41.9%) or senior high school education (34.3%). Over one-third (38.0%) had worked for 6 months to 3 years, and 24.3% had 4–5 years of work experience. Regarding income, 38.8% earned between RMB 3,001 and 4,000 monthly, and 41.8% reported working more than 61 h per week. Approximately 44.0% often paid attention to their own mental health, and 25.4% frequently participated in psychological training. Most participants reported no organic disease (92.4%), and the largest group cared for partially dependent older adults (43.5%), see Table 1.

**Table 1:** Demographic characteristics of the nursing home caregivers (*N* = 1,341).

Variable	*n*	%
City regions		
Northern Henan	334	24.9
Southern Henan	115	8.6
Western Henan	380	28.3
Eastern Henan	62	4.6
Central Henan	450	33.6
Types of nursing homes		
Urban private nursing homes	621	46.3
Urban public nursing homes	417	31.1
Rural private nursing homes	143	10.7
Rural public nursing homes	160	11.9
Gender		
Men	327	24.4
Women	1,014	75.6
Age (years)		
≤20	7	0.5
21–30	51	3.8
31–40	131	9.8
41–50	380	28.3
51–60	625	46.6
≥61	147	11
Educational level		
Primary school and below	167	12.5
Junior high school	562	41.9
Senior high school	460	34.3
Undergraduate	139	10.4
Master’s degree and above	13	1
Marital status		
Unmarried	58	4.3
Married	1,213	90.5
Divorced	70	5.2
Years of working experience		
3–6 months	150	11.2
6 months-3 years	510	38
4–5 years	326	24.3
6–10 years	185	13.8
10–15 years	77	5.7
≥15 years	93	6.9
Monthly income (ť)		
≤2,000	117	8.7
2,001-3,000	311	23.2
3,001-4,000	520	38.8
4,001-5,000	228	17
5,001-6,000	97	7.2
6,001-8,000	50	3.7
≥8,001	18	1.3
Working hours (per week)		
≤30 h	117	8.7
31–40 h	130	9.7
41–50 h	224	16.7
51–60 h	310	23.1
≥61 h	560	41.8
Night shift (per month)		
No	305	22.7
1–5	219	16.3
6–10	212	15.8
10–15	356	26.5
≥15	249	18.6
Paying attention to own mental health		
No	145	10.8
Rarely	257	19.2
Sometimes	349	26
Often	590	44
Participation in psychological training		
No	295	22.0
Rarely	324	24.2
Sometimes	382	28.5
Often	340	25.4
Organic diseases		
No	1,239	92.4
One disease	87	6.5
2–3 diseases	10	0.7
4 or more diseases	5	0.4
Type of older adult care		
Self-care	494	36.8
Partially dependent	584	43.5
Fully dependent	147	11
Special care	116	8.7

### 3.2 Prevalence of depression, anxiety and stress

The mean SAS score was 39.31 ± 8.39, with 89.2% of caregivers showing no anxiety, while 9.0% had mild anxiety, and only 1.8% experienced moderate-to-severe anxiety. The mean SDS score was 44.93 ± 11.22, with 65.2% having no depression, 34.8% had depression, 21.3% mild depression, and 13.5% moderate-to-severe depression. The mean PSS-10 score was 12.92 ± 6.01; half of the caregivers (50.4%) reported very low stress, 32.4% low to moderate stress, and 17.2% moderate-to-high or high stress (Table 2).

**Table 2:** Distribution of anxiety, depression, and stress levels among nursing home caregivers (*N* = 1,341).

Scale	Mean ± SD (min–max)	Category	*n*	%
SAS (Anxiety)	39.31 ± 8.39 (25–73)	No anxiety (<50)	1,196	89.2
		Mild anxiety (50–59)	121	9
		Moderate anxiety (60–69)	21	1.6
		Severe anxiety (70–100)	3	0.2
SDS (Depression)	44.93 ± 11.22 (25–78)	No depression (<50)	874	65.2
		Mild depression (50–59)	286	21.3
		Moderate depression (60–69)	173	12.9
		Severe depression (70–100)	8	0.6
PSS-10 (Stress)	12.92 ± 6.01 (0–34)	Very low stress level (0–13)	676	50.4
		Low to moderate stress level (14–18)	434	32.4
		Moderate to high stress level (19–25)	208	15.5
		High stress level (26–40)	23	1.7

### 3.3 Bivariate factors associated with anxiety, depression, and stress

Bivariate analyses showed that city region, type of nursing home, age, educational level, marital status, monthly income, working hours, night shifts, attention to mental health, participation in psychological training, and presence of organic diseases were significantly associated with anxiety, depression, and stress (all *p* < 0.05). Type of older adult care was significantly related to depression and stress but not anxiety. In contrast, gender and years of working experience showed no significant associations with any of the three outcomes (all *p* > 0.05), see Table 3.

**Table 3:** Univariate analysis of sociodemographic characteristics associated with anxiety, depression, and stress (*N* = 1,341).

Variables	Anxiety		Depression		Stress	
	Mean (SD)	*t/F(P)*	Mean (SD)	*t/F(P)*	Mean (SD)	*t/F(P)*
City regions		10.492 (0.001)		10.492 (0.001)		11.776 (0.001)
Northern Henan	40.46 (8.51)		46.23 (10.67)		14.25 (5.97)	
Southern Henan	41.76 (8.24)		46.53 (11.31)		13.90 (6.19)	
Western Henan	38.34 (7.33)		45.37 (10.30)		12.65 (5.46)	
Eastern Henan	42.90 (8.38)		47.94 (11.35)		14.71 (5.84)	
Central Henan	38.17 (8.83)		42.78 (11.99)		11.65 (6.19)	
Type of nursing home		3.343 (0.019)		3.343 (0.019)		2.933 (0.032)
Urban private NH	38.74 (8.64)		44.46 (11.51)		12.60 (6.30)	
Urban public NH	39.42 (8.22)		44.43 (11.56)		12.80 (5.71)	
Rural private NH	41.14 (8.29)		47.94 (10.29)		14.17 (5.92)	
Rural public NH	39.62 (7.76)		45.38 (11.06)		13.31 (5.62)	
Gender		1.456 (0.146)		−1.553 (0.121)		−1.070 (0.285)
Men	39.90 (8.82)		44.10 (11.31)		12.61 (6.09)	
Women	39.12 (8.25)		45.20 (11.18)		13.02 (5.99)	
Age (years)		11.443 (0.001)		9.770 (0.001)		10.797 (0.001)
≤20	49.14 (6.89)		52.14 (13.52)		16.57 (3.41)	
21–30	44.10 (8.97)		51.35 (11.24)		15.78 (5.53)	
31–40	42.20 (7.96)		49.05 (11.19)		15.44 (4.70)	
41–50	39.57 (7.98)		45.11 (11.09)		13.19 (5.93)	
51–60	38.48 (8.39)		43.66 (10.85)		12.21 (6.14)	
≥61	37.50 (8.23)		43.63 (11.42)		11.76 (6.00)	
Educational level		8.925 (0.001)		4.054 (0.003)		6.107 (0.001)
Primary school and below	37.03 (8.66)		45.10 (10.60)		11.77 (7.08)	
Junior high school	39.18 (7.87)		44.70 (10.91)		12.86 (6.10)	
Senior high school	39.43 (8.49)		44.43 (11.41)		12.83 (5.66)	
Undergraduate	41.40 (8.72)		46.30 (11.86)		14.27 (5.04)	
Master’s degree and above	47.92 (9.77)		56.08 (13.04)		18.39 (3.95)	
Marital status		15.272 (0.001)		11.414 (0.001)		7.325 (0.001)
Unmarried	45.21(9.87)		51.69 (11.39)		15.77 (5.85)	
Married	39.05(8.21)		44.57 (11.06)		12.83 (5.94)	
Divorced	39.04(8.61)		45.54 (12.21)		12.10 (6.92)	
Years of working experience		2.198 (0.052)		1.914 (0.089)		1.030 (0.399)
3–6 months	39.25 (8.39)		46.57 (10.23)		13.09 (5.58)	
6 months-3 years	39.30 (8.30)		44.93 (11.41)		13.19 (6.09)	
4–5 years	38.85 (8.23)		44.78 (11.42)		12.39 (5.96)	
6–10 years	39.02 (8.13)		43.84 (11.13)		13.00 (6.04)	
10–15 years	38.90 (9.65)		42.97 (10.53)		12.23 (6.17)	
≥15 years	42.00 (8.63)		46.66 (11.47)		13.33 (6.33)	
Monthly income (ť)		3.505 (0.002)		3.192 (0.004)		4.609 (0.001)
≤2,000	41.34 (8.32)		47.97 (11.26)		14.58 (5.85)	
2.001–3,000	39.72 (8.69)		45.86 (11.61)		13.59 (6.46)	
3,001-4,000	38.81 (8.06)		44.43 (10.63)		12.48 (5.82)	
4,001-5,000	38.61 (8.29)		44.28 (11.40)		12.69 (5.86)	
5,001-6,000	38.86 (9.19)		42.45 (11.38)		11.15 (5.50)	
6,001-8,000	39.16 (7.46)		43.96 (10.66)		12.90 (6.22)	
≥8,001	45.39 (8.89)		48.06 (14.63)		15.39 (4.97)	
Working hours (per week)		9.721 (0.001)		12.639 (0.001)		8.364 (0.001)
≤30 h	40.80 (7.55)		47.00 (11.50)		13.87 (5.52)	
31–40 h	42.62 (7.51)		50.33 (11.05)		15.22 (5.51)	
41–50 h	39.65 (8.09)		44.95 (11.45)		12.90 (5.43)	
51–60 h	39.48 (8.13)		45.16 (10.80)		13.09 (5.82)	
≥61 h	38.00 (8.75)		43.12 (10.89)		12.09 (6.39)	
Night shift (per month)		8.499 (0.001)		5.967 (0.001)		17.836 (0.001)
No	40.85 (7.80)		46.78 (11.55)		14.62 (5.43)	
1–5	40.34 (8.32)		46.64 (11.91)		13.93 (5.65)	
6–10	39.39 (7.82)		44.54 (10.33)		12.92 (5.63)	
10–15	37.34 (8.26)		43.29 (10.65)		10.98 (5.83)	
≥15	39.27 (9.29)		43.86 (11.28)		12.71 (6.77)	
Paying attention to own mental health		21.436 (0.001)		21.729 (0.001)		25.348 (0.001)
No	37.12 (8.77)		43.78 (10.99)		11.37 (6.63)	
Rarely	41.84 (9.14)		48.66 (11.41)		14.70 (6.28)	
Sometimes	40.80 (8.51)		46.57 (10.65)		14.24 (5.52)	
Often	37.87 (7.42)		42.62 (10.96)		11.73 (5.65)	
Participation in psychological training		7.602 (0.001)		14.268 (0.001)		14.823 (0.001)
No	39.20 (8.51)		45.04 (10.97)		13.39 (6.23)	
Rarely	40.97 (9.27)		48.07 (10.77)		14.25 (6.34)	
Sometimes	39.26 (7.81)		44.29 (11.15)		12.88 (5.52)	
Often	37.89 (7.78)		42.58 (11.30)		11.27 (5.68)	
Organic diseases		38.998 (0.001)		16.441 (0.001)		20.836 (0.001)
No	38.64 (7.96)		44.35 (11.09)		12.56 (5.91)	
One disease	47.01 (8.79)		51.98 (10.29)		17.32 (5.50)	
2–3 diseases	47.00 (12.47)		48.10 (11.10)		15.20 (5.37)	
4 or more diseases	55.40 (10.14)		60.20 (5.81)		20.20 (6.57)	
Type of older adult care		2.134 (0.094)		5.850 (0.001)		2.919 (0.033)
Self-care	39.98 (8.14)		46.57 (10.78)		13.39 (5.74)	
Partially dependent	38.70 (8.38)		43.7 (11.11)		12.53 (5.91)	
Fully dependent	39.31 (9.69)		44.39 (12.02)		13.47 (7.20)	
Special care	39.59 (7.59)		44.49 (11.86)		12.11 (5.88)	

### 3.4 Multivariable factors associated with anxiety, depression, and stress

Multivariable logistic regression analyses identified several independent factors associated with anxiety, depression, and perceived stress among nursing home caregivers (Tables 4–6).

**Table 4:** Multivariable logistic regression for influencing factors of anxiety.

Variables	Prevalence OR	95% CI	*p*-value
City regions			
Northern Henan			
Southern Henan	0.946	0.458–1.951	0.880
Western Henan	0.513	0.284–0.929	0.028
Eastern Henan	1.628	0.681–3.893	0.273
Central Henan	0.962	0.581–1.592	0.881
Type of nursing home			
Urban private NH			
Urban public NH	0.974	0.606–1.565	0.913
Rural private NH	1.030	0.539–1.971	0.928
Rural public NH	0.671	0.328–1.370	0.273
Age (years)			
≤20			
21–30	2.094	0.282–15.555	0.470
31–40	2.157	0.235–19.825	0.497
41–50	1.174	0.133–10.389	0.885
51–60	0.913	0.105–7.980	0.935
≥61	0.516	0.055–4.843	0.562
Educational level			
Primary school and below			
Junior high school	1.095	0.536–2.238	0.804
Senior high school	1.671	0.794–3.518	0.176
Undergraduate	1.389	0.540–3.577	0.496
Master’s degree and above	6.924	1.388–34.544	0.018
Marital status			
Unmarried			
Married	0.465	0.156–1.386	0.170
Divorced	0.615	0.156–2.427	0.487
Monthly income (ť)			
≤2,000			
2.001–3,000	0.609	0.297–1.248	0.175
3,001–4,000	0.482	0.239–0.976	0.043
4,001–5,000	0.525	0.231–1.193	0.124
5,001–6,000	0.490	0.186–1.289	0.148
6,001–8,000	0.119	0.023–0.613	0.011
≥8,001	1.703	0.405–7.165	0.467
Working hours (per week)			
≤30 h			
31–40 h	1.511	0.607–3.761	0.375
41–50 h	1.431	0.601–3.407	0.418
51–60 h	1.795	0.776–4.152	0.172
≥61	1.455	0.636–3.329	0.375
Night shift (per month)			
No			
1–5	0.731	0.394–1.355	0.319
6–10	0.816	0.430–1.549	0.534
10–15	0.657	0.361–1.197	0.170
≥15	1.179	0.618–2.251	0.618
Paying attention to own mental health			
No			
Rarely	1.219	0.582–2.552	0.600
Sometimes	1.083	0.517–2.266	0.833
Often	0.460	0.203–1.045	0.064
Participation in psychological training			
No			
Rarely	1.360	0.796–2.324	0.261
Sometimes	0.857	0.480–1.529	0.600
Often	0.800	0.371–1.724	0.568
Organic diseases			
No			
One disease	6.153	3.573–10.596	0.001
2–3 diseases	7.139	1.521–33.501	0.013
4 or more diseases	9.385	1.116–78.927	0.039

For anxiety, significant predictors included city region, educational level, monthly income, and presence of chronic diseases. Caregivers with a master’s degree or above had significantly higher odds of anxiety (OR = 6.924, 95% CI = 1.388–34.544), whereas those with moderate income levels of 3,001–4,000 RMB (OR = 0.482, 95% CI = 0.239–0.976) and 6,001–8,000 RMB (OR = 0.119, 95% CI = 0.023–0.613) showed lower odds. The presence of chronic diseases increased the likelihood of anxiety in a dose–response manner, with one disease (OR = 6.153, 95% CI = 3.573–10.596), 2–3 diseases (OR = 7.139, 95% CI = 1.521–33.501), and 4 or more diseases (OR = 9.385, 95% CI = 1.116–78.927) all associated with higher odds.

For depression, significant factors included city region, type of nursing home, educational level, working hours, night shifts, attention to mental health, participation in psychological training, and presence of chronic diseases. Higher education (undergraduate: OR = 0.509, 95% CI = 0.269–0.965) and income (3,001–4,000 RMB: OR = 0.518, 95% CI = 0.320–0.839; 5,001–6,000 RMB: OR = 0.329, 95% CI = 0.167–0.650) were protective factors, whereas employment in rural private nursing homes (OR = 1.617, 95% CI = 1.051–2.488), limited attention to mental health (rarely: OR = 1.949, 95% CI = 1.156–3.288), infrequent participation in psychological training (rarely: OR = 1.668, 95% CI = 1.137–2.446), and presence of chronic diseases (one disease: OR = 3.108, 95% CI = 1.910–5.056) were associated with increased odds of depression.

Perceived stress was independently associated with city region, type of nursing home, monthly income, working hours, night shifts, attention to mental health, participation in psychological training, and presence of chronic diseases. Higher monthly income (3,001–4,000 RMB: OR = 0.467, 95% CI = 0.287–0.759; 5,001–6,000 RMB: OR = 0.229, 95% CI = 0.117–0.448), longer working hours (≥61 h/week: OR = 0.596, 95% CI = 0.364–0.977), and more frequent night shifts (6–10/month: OR = 0.595, 95% CI = 0.395–0.895; 10–15/month: OR = 0.326, 95% CI = 0.222–0.479; ≥15/month: OR = 0.492, 95% CI = 0.320–0.755) were associated with lower levels of stress, while employment in urban public (OR = 1.380, 95% CI = 1.022–1.864) or rural private nursing homes (OR = 1.657, 95% CI = 1.075–2.553), limited attention to mental health (sometimes: OR = 1.833, 95% CI = 1.121–2.997), infrequent participation in psychological training (rarely: OR = 1.668, 95% CI = 1.137–2.446), and presence of chronic diseases (one disease: OR = 3.732, 95% CI = 2.144–6.494) were linked to an increased risk of stress.

Overall, regional disparities, income, education level, chronic disease burden, and engagement in mental health practices emerged as independent predictors across all three psychological outcomes.

**Table 5:** Multivariable logistic regression for influencing factors of depression.

Variables	Prevalence OR	95% CI	*p*-value
City regions			
Northern Henan			
Southern Henan	0.638	0.382–1.067	0.087
Western Henan	0.633	0.434–0.923	0.018
Eastern Henan	0.977	0.508–1.879	0.945
Central Henan	0.625	0.442–0.884	0.008
Type of nursing home			
Urban private NH			
Urban public NH	1.190	0.870–1.628	0.276
Rural private NH	1.617	1.051–2.488	0.029
Rural public NH	0.992	0.640–1.539	0.973
Age (years)			
≤20			
21–30	1.446	0.203–10.282	0.713
31–40	1.347	0.167–10.887	0.780
41–50	0.698	0.089–5.484	0.732
51–60	0.509	0.065–4.001	0.521
≥61	0.450	0.056–3.647	0.455
Educational level			
Primary school and below			
Junior high school	1.005	0.646–1.565	0.982
Senior high school	0.879	0.549–1.406	0.590
Undergraduate	0.509	0.269–0.965	0.039
Master’s degree and above	2.673	0.619–11.548	0.188
Marital status			
Unmarried			
Married	0.597	0.238–1.498	0.272
Divorced	0.912	0.314–2.654	0.866
Monthly income (ť)			
≤2,000			
2.001–3,000	0.683	0.420–1.111	0.125
3,001–4,000	0.518	0.320–0.839	0.007
4,001–5,000	0.771	0.448–1.327	0.347
5,001–6,000	0.329	0.167–0.650	0.001
6,001–8,000	0.584	0.262–1.302	0.189
≥8,001	1.515	0.448–5.120	0.504
Working hours (per week)			
≤30 h			
31–40 h	1.077	0.618–1.877	0.794
41–50 h	0.711	0.425–1.191	0.195
51–60 h	0.682	0.416–1.116	0.128
≥61	0.459	0.280–0.754	0.002
Night shift (per month)			
No			
1–5	1.257	0.842–1.877	0.263
6–10	0.906	0.601–1.366	0.637
10–15	0.584	0.394–0.865	0.007
≥15	0.788	0.504–1.233	0.297
Paying attention to own mental health			
No			
Rarely	1.949	1.156–3.288	0.012
Sometimes	1.566	0.933–2.630	0.090
Often	0.842	0.490–1.446	0.533
Participation in psychological training			
No			
Rarely	1.668	1.137–2.446	0.009
Sometimes	1.182	0.797–1.754	0.405
Often	1.164	0.735–1.843	0.517
Organic diseases			
No			
One disease	3.108	1.910–5.056	0.001
2–3 diseases	5.310	0.321–5.310	0.321
4 or more diseases	3.391	0.167–4.031	0.969
Type of older adult care			
Self-care			
Partially dependent	0.691	0.506–0.944	0.020
Fully dependent	1.111	0.685–1.802	0.670
Special care	1.397	0.849–2.297	0.188

**Table 6:** Multivariable logistic regression for influencing factors of Perceived Stress.

Variables	Prevalence OR	95% CI	*p*-value
City regions			
Northern Henan	Reference	Reference	Reference
Southern Henan	0.501	0.301–0.834	0.008
Western Henan	0.538	0.371–0.779	0.001
Eastern Henan	0.450	0.450–1.638	0.858
Central Henan	0.449	0.321–0.627	0.001
Type of nursing home			
Urban private NH	Reference	Reference	Reference
Urban public NH	1.380	1.022–1.864	0.035
Rural private NH	1.657	1.075–2.553	0.022
Rural public NH	1.119	0.734–1.708	0.601
Age (years)			
≤20	Reference	Reference	Reference
21–30	0.681	0.055–8.416	0.765
31–40	0.644	0.047–8.886	0.742
41–50	0.336	0.025–4.522	0.411
51–60	0.273	0.020–3.679	0.328
≥61	0.212	0.015–2.935	0.247
Educational level			
Primary school and below	Reference	Reference	Reference
Junior high school	1.503	0.980–2.305	0.062
Senior high school	1.206	0.765–1.899	0.420
Undergraduate	0.903	0.486–1.678	0.747
Master’s degree and above	2.027	0.093–5.940	0.998
Marital status			
Unmarried	Reference	Reference	Reference
Married	1.221	0.469–3.179	0.682
Divorced	1.091	0.362–3.287	0.877
Monthly income (ť)			
≤2,000	Reference	Reference	Reference
2.001–3,000	0.629	0.383–1.032	0.067
3,001-4,000	0.467	0.287–0.759	0.002
4,001-5,000	0.646	0.375–1.111	0.114
5,001-6,000	0.229	0.117–0.448	0.001
6,001-8,000	0.671	0.306–1.471	0.319
≥8,001	0.895	0.239–3.353	0.869
Working hours (per week)			
≤30 h	Reference	Reference	Reference
31–40 h	1.100	0.616–1.962	0.748
41–50 h	0.755	0.450–1.266	0.286
51–60 h	0.872	0.531–1.432	0.588
≥61	0.596	0.364–0.977	0.040
Night shift (per month)			
No	Reference	Reference	Reference
1–5	0.676	0.451–1.015	0.059
6–10	0.595	0.395–0.895	0.013
10–15	0.326	0.222–0.479	0.001
≥15	0.492	0.320–0.755	0.001
Paying attention to own mental health			
No	Reference	Reference	Reference
Rarely	1.601	0.972–2.635	0.064
Sometimes	1.833	1.121–2.997	0.016
Often	1.012	0.611–1.676	0.964
Participation in psychological training			
No	Reference	Reference	Reference
Rarely	1.173	0.803–1.715	0.409
Sometimes	0.770	0.528–1.123	0.174
Often	0.545	0.353–0.842	0.006
Organic diseases			
No	Reference	Reference	Reference
One disease	3.732	2.144–6.494	0.001
2–3 diseases	1.246	0.280–5.541	0.773
4 or more diseases	2.459	0.745–1.641	0.989
Type of older adult care			
Self-care	Reference	Reference	Reference
Partially dependent	0.993	0.732–1.346	0.962
Fully dependent	1.450	0.909–2.312	0.119
Special care	0.985	0.601–1.612	0.951

## 4 Discussion

This multicenter study examined the prevalence of depression, anxiety, and perceived stress, as well as associated factors, among nursing home caregivers in Henan Province, China. The findings revealed that depression was the most prevalent psychological problem, followed by stress, whereas anxiety was relatively uncommon. Multiple sociodemographic, occupational, health-related, and behavioral factors showed independent associations with these outcomes. These results add new empirical evidence to the body of research on caregivers’ mental health and highlight priority areas for intervention and policy development.

In this sample, 34.8% of caregivers experienced depression, 49.8% reported varying levels of stress, and only 10.8% presented with anxiety. Depression and stress emerged as the dominant psychological concerns, while anxiety prevalence was notably lower than in studies conducted among Japanese and Spanish caregivers (18, 19). One plausible explanation lies in the age distribution of this sample, as 56.6% of participants were aged 51 years or older. According to psychosocial development theory, individuals in middle and late adulthood generally demonstrate stronger emotional regulation and psychological stability under stress (38). Consistent with prior research, age was negatively correlated with anxiety, indicating that older caregivers tend to report lower anxiety levels (39, 40).

The prevalence of depression in this study is consistent with findings from several domestic (12, 29) and international studies (18, 19, 41). For example, Chen, Cao (29) reported a prevalence of 36.3% in Shandong Province, while Santos-Orlandi, Brigola (42) found a rate of 29.6% among Brazilian caregivers. Stress prevalence was also comparable to international reports (19, 20). Collectively, these findings underscore the global significance of depression and stress as occupational health challenges in long-term care facilities.

Marked regional disparities were identified, with caregivers in the southern, western, and central regions reporting better mental health than those in the northern and eastern regions. This finding aligns with evidence that inequalities in socioeconomic conditions and healthcare resources influence mental health outcomes (43, 44). Education showed complex effects. Postgraduate education was linked to higher anxiety, which may reflect role mismatch and career frustration, whereas a bachelor’s degree appeared protective against depression, consistent with research connecting health literacy to psychological resilience (45, 46). Income demonstrated a U-shaped relationship, where moderate income was most protective, while both low- and high-income groups experienced greater psychological distress. This trend is consistent with broader socioeconomic health research (47, 48). Although not significant in multivariate models, bivariate analyses suggested that unmarried caregivers were more vulnerable, underscoring the protective influence of social support (25, 49).

Institutional characteristics also significantly influenced mental health. Caregivers in rural private nursing homes reported higher levels of depression and stress, aligning with prior findings that underfunded institutions impose heavier burdens on staff (44). Interestingly, longer working hours and more frequent night shifts were associated with lower levels of depression and stress, contrary to most previous studies (23, 29). This paradoxical pattern may reflect the “healthy worker effect,” income-related stress buffering, or cultural adaptation to shift work (50). Longitudinal studies are needed to verify these explanations.

Physical illness emerged as a strong predictor of psychological distress, with risk increasing alongside the number of chronic conditions. This finding is consistent with prior research showing that physical morbidity contributes to depression, anxiety, and stress through both biological and psychosocial pathways (23, 51). Caregivers with chronic diseases face compounded burdens, suggesting the need for integrated physical and psychological support.

Several modifiable factors were found to be protective. Caregivers who regularly attended to their mental health and participated in psychological training reported lower risks of depression and stress. This finding aligns with evidence supporting the effectiveness of psychoeducation, mindfulness, and stress management interventions (52–54). However, only one-quarter of caregivers in this study reported regular participation in psychological training, highlighting substantial gaps in institutional support.

### 4.1 Theoretical framing and inferential model

From a theoretical perspective, the current findings can be interpreted through an inferential model based on the ABC Theory of Emotion (55) and the stress and coping theory (56). Anchored in these frameworks, our results can be organized into a concise inferential pathway. Contextual and individual exposures, such as region, facility type, education, income, and chronic disease, constitute the “activating events” (A) that shape caregivers’ cognitive appraisal (B), which is operationalized here as perceived stress. These appraisals then give rise to “emotional consequences” (C), observed as depression and anxiety. This structure helps explain why predictors that elevate moderate-to-high perceived stress, such as lower income, rural private facilities, and chronic disease, also increase risks of depression and anxiety, whereas resource variables including attention to mental health and psychological training are broadly protective. In other words, training and proactive mental health attention likely enhance adaptive appraisal and coping, thereby attenuating the A → B → C cascade. While causal inference is limited by the cross-sectional design, this theory-grounded model provides a foundation for future longitudinal and structural equation modeling studies to quantify indirect effects (exposure → perceived stress → symptoms) and to examine whether workplace training strengthens resilience pathways within the aging care workforce.

### 4.2 Implications

These findings have important implications. Clinically, routine screening for depression and stress should be prioritized, particularly among caregivers with chronic illnesses and those working in resource-limited institutions. At the policy level, efforts are needed to improve working conditions, ensure fair compensation, and expand access to psychological training. From a research perspective, longitudinal and interventional studies are essential to clarify causal pathways and evaluate the effectiveness of workplace-based interventions.

### 4.3 Strengths and limitations

This study benefits from a large, multi-center sample and the use of validated scales, providing robust evidence on an under-researched population. Nonetheless, several limitations must be acknowledged. The cross-sectional design precludes causal inference, self-reported measures may introduce bias, and findings from Henan Province may not generalize to other regions or countries. Future research should adopt longitudinal and mixed-method approaches across diverse settings.

## 5 Conclusion

Depression and stress are significant psychological concerns among nursing home caregivers in China, influenced by regional, educational, economic, institutional, health-related, and behavioral factors. Addressing these challenges requires coordinated clinical, organizational, and policy-level interventions to promote caregivers’ mental well-being and ensure the sustainability of older adult care services in an aging society.

## Data Availability

The raw data supporting the conclusions of this article will be made available by the authors, without undue reservation.
