# Supplementary material for: Prevalence and factors associated with depression, anxiety and stress among nursing home caregivers in China: a multi-center cross-sectional study
Source: Front Public Health. 2025 Nov 3;13:1690840. doi: 10.3389/fpubh.2025.1690840 (PMC12620222; doi:10.3389/fpubh.2025.1690840)
Supplement: Supplementary file 1 [file Supplementary_file_1.docx]

**Supplementary File**

**1. Hosmer–Lemeshow goodness-of-fit test**

In this study, sociodemographic variables that were significantly associated with anxiety (SAS), depression (SDS), and perceived stress (PSS-10) in univariate analyses were subsequently entered into the corresponding multivariable logistic regression models. The Hosmer–Lemeshow goodness-of-fit test was used to assess model adequacy. The results showed Chi-square values of 9.226 (df = 8, p = 0.324) for SAS, 9.334 (df = 8, p = 0.315) for SDS, and 6.987 (df = 8, p = 0.538) for PSS. As all p values exceeded 0.05, the models were considered to have good fits, indicating no significant differences between predicted probabilities and observed outcomes. These findings suggest that the multivariable logistic regression models were robust in capturing the relationships between sociodemographic characteristics and psychological distress among nursing home caregivers (Table S1, Table S2, Table S3).

Table S1. Hosmer–Lemeshow goodness-of-fit test for the multivariable logistic regression model of sociodemographic factors and anxiety (SAS) (N = 1,341).

| Step | Chi-square | df | Sig. |
| --- | --- | --- | --- |
| SAS | 9.226 | 8 | 0.324 |

Table S2. Hosmer–Lemeshow goodness-of-fit test for the multivariable logistic regression model of sociodemographic factors and depression (SDS) (N = 1,341).

| Step | Chi-square | df | Sig. |
| --- | --- | --- | --- |
| SDS | 9.334 | 8 | 0.315 |

Table S3. Hosmer–Lemeshow goodness-of-fit test for the multivariable logistic regression model of sociodemographic factors and perceived stress (PSS-10) (N = 1,341).

| Step | Chi-square | df | Sig. |
| --- | --- | --- | --- |
| PSS-10 | 6.987 | 8 | 0.538 |

**2. Collinearity Diagnostics**

Before performing multiple logistic regression analyses, multiple linear regression analyses were conducted to assess potential multicollinearity. Collinearity diagnostics for the three models examining the effects of sociodemographic and work-related factors on anxiety (SAS scores), depression (SDS scores), and perceived stress (PSS scores) indicated no multicollinearity, as all variance inflation factors (VIFs) were below 2. This confirmed that the predictors were not highly correlated and each independently contributed to the respective outcomes (Table S4, Table S5, Table S6).

Table S4. Multiple Linear Regression Coefficients and Collinearity Diagnostics for Anxiety (SAS Scores)

| Variables | B | Std. Error | Beta | t | p | Tolerance | VIF |
| --- | --- | --- | --- | --- | --- | --- | --- |
| Constant | 43.701 | 2.266 |  | 19.287 | 0.000 |  |  |
| City regions | -0.472 | 0.137 | -0.088 | -3.439 | 0.001 | 0.975 | 1.026 |
| **Type of nursing home** | 0.387 | 0.221 | 0.047 | 1.753 | 0.080 | 0.895 | 1.117 |
| Age (years) | -1.231 | 0.246 | -0.143 | -4.998 | 0.000 | 0.776 | 1.288 |
| Educational level | 0.628 | 0.288 | 0.065 | 2.181 | 0.029 | 0.712 | 1.405 |
| Marital status | -0.319 | 0.735 | -0.012 | -0.434 | 0.664 | 0.874 | 1.144 |
| Monthly income | -0.079 | 0.183 | -0.012 | -0.435 | 0.663 | 0.851 | 1.175 |

Table S4 (continued)

| Working hours (per week) | -0.659 | 0.185 | -0.103 | -3.555 | 0.000 | 0.764 | 1.308 |
| --- | --- | --- | --- | --- | --- | --- | --- |
| Night shift (per month) | -0.129 | 0.167 | -0.022 | -0.774 | 0.439 | 0.777 | 1.287 |
| Paying attention to own mental health | -0.550 | 0.260 | -0.068 | -2.116 | 0.035 | 0.626 | 1.596 |
| Participation in psychological training | -0.465 | 0.244 | -0.061 | -1.908 | 0.057 | 0.635 | 1.576 |
| Organic diseases | 6.410 | 0.619 | 0.265 | 10.360 | 0.000 | 0.979 | 1.021 |

Note. Dependent variable: SAS Scores.

Table S5. Multiple Linear Regression Coefficients and Collinearity Diagnostics for Depression (SDS Scores)

| Predictor | B | Std. Error | Beta | t | p | Tolerance | VIF |
| --- | --- | --- | --- | --- | --- | --- | --- |
| Constant | 59.649 | 3.091 |  | 19.295 | 0.000 |  |  |
| City regions | -0.732 | 0.187 | -0.102 | -3.912 | 0.000 | 0.974 | 1.026 |
| **Type of nursing home** | 0.377 | 0.302 | 0.034 | 1.245 | 0.213 | 0.884 | 1.131 |
| Age (years) | -1.664 | 0.337 | -0.145 | -4.945 | 0.000 | 0.773 | 1.294 |
| Educational level | -0.259 | 0.393 | -0.020 | -0.661 | 0.509 | 0.711 | 1.407 |
| Marital status | 0.170 | 1.001 | 0.005 | 0.170 | 0.865 | 0.874 | 1.144 |
| Monthly income | -0.316 | 0.249 | -0.035 | -1.267 | 0.205 | 0.849 | 1.178 |
| Working hours (per week) | -1.171 | 0.256 | -0.137 | -4.581 | 0.000 | 0.745 | 1.341 |
| Night shift (per month) | -0.353 | 0.231 | -0.045 | -1.528 | 0.127 | 0.753 | 1.329 |
| Paying attention to own mental health | -1.180 | 0.356 | -0.108 | -3.310 | 0.001 | 0.619 | 1.615 |
| Participation in psychological training | -0.650 | 0.332 | -0.063 | -1.956 | 0.051 | 0.634 | 1.576 |
| Organic diseases | 5.530 | 0.843 | 0.171 | 6.556 | 0.000 | 0.979 | 1.021 |
| Type of older adult care | 0.178 | 0.345 | 0.014 | 0.515 | 0.607 | 0.861 | 1.162 |

Note. Dependent variable: SDS Scores.

Table S6. Multiple Linear Regression Coefficients and Collinearity Diagnostics for Perceived Stress (PSS Scores)

| Predictor | B | Std. Error | Beta | *t* | *p* | Tolerance | VIF |
| --- | --- | --- | --- | --- | --- | --- | --- |
| Constant | 19.166 | 1.630 |  | 11.759 | 0.000 |  |  |
| City regions | -0.547 | 0.099 | -0.142 | -5.548 | 0.000 | 0.974 | 1.026 |
| **Type of nursing home** | 0.268 | 0.159 | 0.045 | 1.681 | 0.093 | 0.884 | 1.131 |
| Age (years) | -0.844 | 0.177 | -0.137 | -4.758 | 0.000 | 0.773 | 1.294 |
| Educational level | 0.305 | 0.207 | 0.044 | 1.474 | 0.141 | 0.711 | 1.407 |
| Marital status | -0.018 | 0.528 | -0.001 | -0.035 | 0.972 | 0.874 | 1.144 |
| Monthly income | -0.217 | 0.131 | -0.045 | -1.650 | 0.099 | 0.849 | 1.178 |
| Working hours (per week) | -0.314 | 0.135 | -0.068 | -2.327 | 0.020 | 0.745 | 1.341 |
| Night shift (per month) | -0.494 | 0.122 | -0.118 | -4.052 | 0.000 | 0.753 | 1.329 |

Table S6 (continued)

| Paying attention to own mental health | -0.164 | 0.188 | -0.028 | -0.871 | 0.384 | 0.619 | 1.615 |
| --- | --- | --- | --- | --- | --- | --- | --- |
| Participation in psychological training | -0.820 | 0.175 | -0.149 | -4.681 | 0.000 | 0.634 | 1.576 |
| Organic diseases | 3.141 | 0.445 | 0.181 | 7.064 | 0.000 | 0.979 | 1.021 |
| Type of older adult care | 0.297 | 0.182 | 0.045 | 1.632 | 0.103 | 0.861 | 1.162 |

Note. Dependent variable: PSS-10 Scores.
